# Induction of fibroblast senescence generates a non-fibrogenic myofibroblast phenotype that differentially impacts on cancer prognosis

**DOI:** 10.18632/aging.101127

**Published:** 2016-12-15

**Authors:** Massimiliano Mellone, Christopher J. Hanley, Steve Thirdborough, Toby Mellows, Edwin Garcia, Jeongmin Woo, Joanne Tod, Steve Frampton, Veronika Jenei, Karwan A. Moutasim, Tasnuva D. Kabir, Peter A Brennan, Giulia Venturi, Kirsty Ford, Nicolas Herranz, Kue Peng Lim, James Clarke, Daniel W. Lambert, Stephen S. Prime, Timothy J. Underwood, Pandurangan Vijayanand, Kevin W. Eliceiri, Christopher Woelk, Emma V. King, Jesus Gil, Christian H. Ottensmeier, Gareth J. Thomas

**Affiliations:** ^1^ Cancer Sciences Unit, Faculty of Medicine, University of Southampton, SO166YD, UK; ^2^ Faculty of Medicine, University of Southampton, Southampton SO166YD, UK; ^3^ Integrated Biosciences, School of Clinical Dentistry, University of Sheffield, Sheffield S102TA, UK; ^4^ Queen Alexandra Hospital, Portsmouth Hospitals NHS Trust, Portsmouth PO63LY, UK; ^5^ MRC Clinical Sciences Centre, Imperial College Faculty of Medicine, Hammersmith Hospital Campus, London W12, UK; ^6^ Cancer Research Initiatives Foundation. Sime Darby Medical Centre, Subang Jaya, Selangor 47500, Malaysia; ^7^ Centre for Clinical and Diagnostic Oral Sciences, Institute of Dentistry, Barts and the London School of Medicine and Dentistry, Queen Mary University of London, London E12AD, UK; ^8^ La Jolla Institute for Allergy & Immunology, La Jolla, California 92037, USA; ^9^ Laboratory for Optical and Computational Instrumentation (LOCI), Department of Biomedical Engineering, University of Madison, Wisconsin, WI 53706, USA

**Keywords:** tumor microenvironment, myofibroblasts, senescence, collagen, extracellular matrix, senescent fibroblasts

## Abstract

Cancer-associated fibroblasts (CAF) remain a poorly characterized, heterogeneous cell population. Here we characterized two previously described tumor-promoting CAF sub-types, smooth muscle actin (SMA)-positive myofibroblasts and senescent fibroblasts, identifying a novel link between the two. Analysis of CAF cultured *ex vivo*, showed that senescent CAF are predominantly SMA-positive; this was confirmed by immunochemistry in head & neck (HNSCC) and esophageal (EAC) cancers. *In vitro*, we found that fibroblasts induced to senesce develop molecular, ultrastructural and contractile features typical of myofibroblasts and this is dependent on canonical TGF-β signaling. Similar to TGF-β1-generated myofibroblasts, these cells secrete soluble factors that promote tumor cell motility. However, RNA-sequencing revealed significant transcriptomic differences between the two SMA-positive CAF groups, particularly in genes associated with extracellular matrix (ECM) deposition and organization, which differentially promote tumor cell invasion. Notably, second harmonic generation imaging and bioinformatic analysis of SMA-positive human HNSCC and EAC showed that collagen fiber organization correlates with poor prognosis, indicating that heterogeneity within the SMA-positive CAF population differentially impacts on survival. These results show that non-fibrogenic, SMA-positive myofibroblasts can be directly generated through induction of fibroblast senescence and suggest that senescence and myofibroblast differentiation are closely linked processes.

## INTRODUCTION

Cancer-associated fibroblasts (CAF) have been shown to promote many, if not all, of the ‘hallmarks of malignancy’ [1]. Despite their tumor-promoting properties, CAF remain a poorly-defined, heterogeneous cell population, possibly reflecting their cell(s) of origin, the tissue in which they develop, and their activation state [1-3]. No single marker reliably identifies all CAF, however, the most commonly analyzed sub-type have a contractile myofibroblastic phenotype, characterized by expression of α-smooth muscle actin (SMA) [4, 5]. While a number of signaling pathways contribute to myofibroblast transdifferentiation, complete transdifferentiation requires both TGF-β1 signaling and mechanotransduction (i.e. increased tissue tension), mediated through Smad/Rho activation [6]. Myofibroblasts have been shown to promote tumor cell invasion and metastasis, and an SMA-positive, myofibroblast-rich stroma is prognostic in several cancers [7-10]. The motility-promoting effects of myofibroblasts result, at least in part, from their contractility and remodeling of collagenous extracellular matrix (ECM) proteins, which serves to generate tissue tension and increased matrix stiffness [5, 11]. ECM remodeling through thickening, linearization, elongation or crosslinking of collagen fibers, is common in cancers, and often found in areas where active cancer cell migration occurs [12-14]. However, recent studies have shown that there is a significant degree of heterogeneity in how CAFs interact with the ECM which influences disease progression [15, 16].

While CAFs are generally associated with having a myofibroblastic phenotype, recent research has identified a number of other CAF phenotypes [17, 18], including senescent CAF [19-21], which may also impact on tumor development and progression [19]. Senescence can be induced by a variety of intracellular and extracellular stimuli, including telomere dysfunction resulting from repeated cell division (replicative senescence) and DNA damage induced through oxidative or genotoxic stress (pre-replicative senescence) [22]. These stimuli induce Ataxia Telangiectasia Mutated kinase (ATM) to activate the DNA-damage response pathway to repair DNA damage. However, in the event of extensive, unrepairable DNA damage, cells undergo permanent growth arrest via p53/p21 or p16/pRB pathways [22]. Emerging evidence suggests that senescent CAF may also be tumor promoting [19-21], acquiring a senescence-associated secretory phenotype (SASP) that creates a permissive microenvironment favoring tumor development [20, 21, 23]. Notably, in fibrotic disease and wound healing, senescence of fibroblasts has been described as a mechanism for limiting fibrosis by suppressing ECM-production [24-26]. Whether fibroblast senescence functions similarly to limit collagen deposition in tumors has yet to be determined, but we have described previously varying ability for collagen fibrogenesis in CAF isolated from esophageal cancer [14].

In this study we analyzed human tumors and CAF cultured *ex vivo*, and found that senescent CAF are mostly SMA-positive. We compared the molecular and functional characteristics of TGF-β1-treated myofibroblasts with fibroblasts induced to senesce through extended culture or various DNA damaging stimuli (irradiation, H_2_O_2_, cisplatin). Unexpectedly we found that, similar to TGF-β1 treatment, induction of fibroblast senescence generates an SMA-positive, contractile cell with molecular and ultrastructural features of a myofibroblast, and this is dependent on canonical TGF-β signaling. Transcriptomic analysis of TGF-β1-treated myofibroblasts and senescent fibroblasts revealed common gene expression related to contractile function, but with mostly divergent gene expression profiles. In particular senescent fibroblasts showed reduced ECM deposition and organization which affected cancer cell invasion. Bioinformatic and second harmonic generation analysis of these cells *in vivo* confirmed that there are significant differences in collagen production and structure within the tumor stroma. Moreover, in cohorts of patients with head & neck (HNSCC) and esophagus (EAC) cancer containing an SMA-positive stroma, the expression of collagen fibril organizing genes and the presence of an organized collagen matrix in the form of elongated collagen fibers is associated with the poorest prognosis. These results suggest that myofibroblast transdifferentiation and senescence are closely linked processes, whereby non-fibrogenic myofibroblasts can be directly generated through induction of fibroblast senescence; this heterogeneity within the SMA-positive CAF population differentially impacts on patient survival.

## RESULTS

### Senescent CAF commonly co-express SMA *ex vivo* and *in vivo*

**Figure 1:**
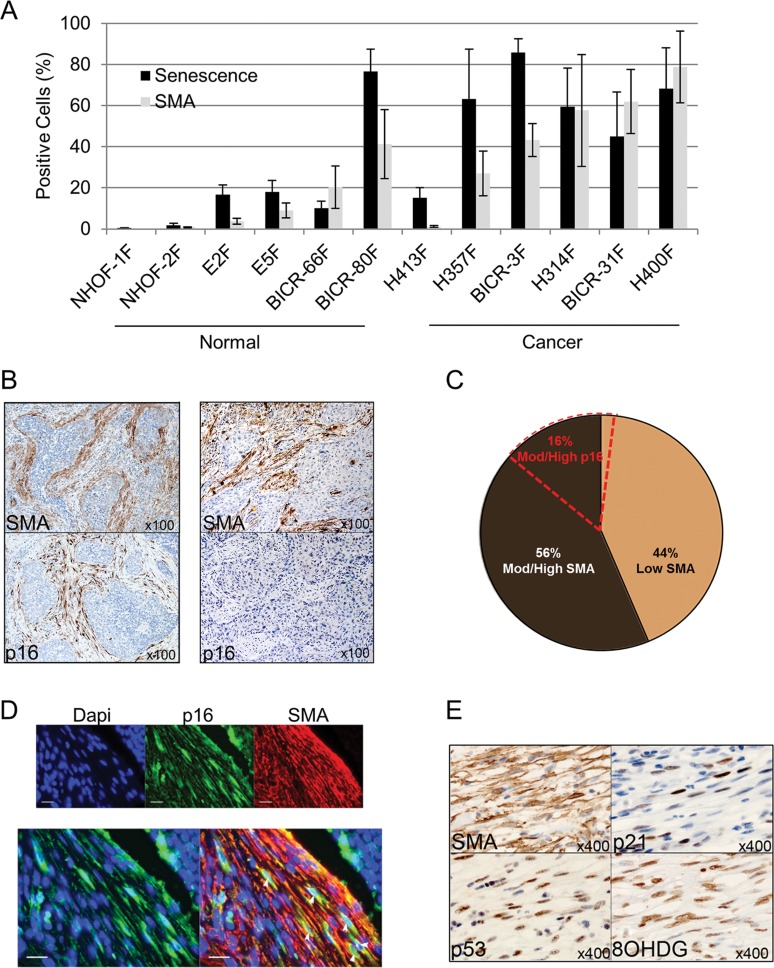
Senescent CAF analyzed *ex vivo* and *in vivo* are predominantly SMA-positive (**A**) Histogram showing percentage of cells positive for senescence-associated (SA)-β-Galactosidase or SMA-positive stress fiber formation in normal oral fibroblasts (POF) and cancer-associated oral fibroblasts (CAF) grown *ex-vivo*. Data are presented as Mean ±SEM from 6 POFs and 6 CAF. (**B**) Representative images of immunohistochemistry on sequential tissue sections of SMA-positive/p16-positive or SMA-positive/p16-negative HNSCC cases. (**C**) Pie chart showing the percentage of HNSCC cases with stromal staining for SMA or p16. (**D**) Representative image of double immunofluorescence staining of a p16-positive/SMA-positive HNSCC case showing co-expression of SMA (red) and p16 (green, white arrows; scale bars represent 25μm). (**E**) Representative immunohistochemistry for SMA and markers of senescence (p53, p21) and oxidative stress (8-OHDG) on sequential tissue sections of HNSCC. See also Supplementary Fig. S2.

To investigate the relationship between myofibroblast differentiation and fibroblast senescence, we cultured primary normal oral fibroblasts and CAF from human head and neck cancers (HNSCC) *ex vivo* and examined co-expression of SMA and SA-β-Gal (Fig. 1A). In normal fibroblasts there was a highly positive correlation between senescence and SMA expression (r^2^=0.82; Supplementary Fig. S1A); this correlation was weaker in CAFs (r^2^=0.32; Supplementary Fig. S1B), possibly suggesting greater heterogeneity in this fibroblast population.

To rule out the possibility of artefacts due to cell culture conditions, these *ex vivo* observations were analyzed further using immunohistochemistry for markers of myofibroblast transdifferentiation (SMA) and senescence (p16) on HNSCC tissues (n=96). p16 and SMA were expressed in similar regions of the stroma (Fig. 1B) and, similar to CAF cultures *ex vivo*, most cases (87%; n=13/15) with p16-positive stromal staining also showed strong/moderate SMA positivity; conversely 76% of cases with strong/moderate SMA positivity did not express stromal p16 (41/54; Fig. 1C; Table 1). Co-expression of p16 and SMA was confirmed by dual immunofluorescence staining (Fig. 1D). Since no single marker identifies senescent cells in tissues, we also examined expression of additional proteins associated with senescence (markers of cell-cycle arrest and oxidative DNA damage; p53, p21 and 8-OHdG respectively [22, 27, 28]) in 10 tumors with p16-positive/SMA-positive stromal cells, and found expression of these senescence markers in similar tissue areas in all cases (Fig. 1E). Similar findings were observed in esophageal cancer (EAC; n=21) with co-expression of SMA/p53/p21 in 2/16 (12.5%) of the SMA-positive cases (Supplementary Fig. S1C and Supplementary Table S1). Thus a subset (ranging from 12.5-24%) of tumors that contain SMA-positive stroma co-express markers of senescence. Conversely senescent CAF usually have an SMA-positive phenotype.

**Table 1:** SMA and p16 positive HNSCC cases

	SMA low	SMA Mod/High
**p16 Mod/High**	2	13
**p16 Low**	40	41

### Induction of fibroblast senescence generates a myofibroblastic phenotype

**Figure 2:**
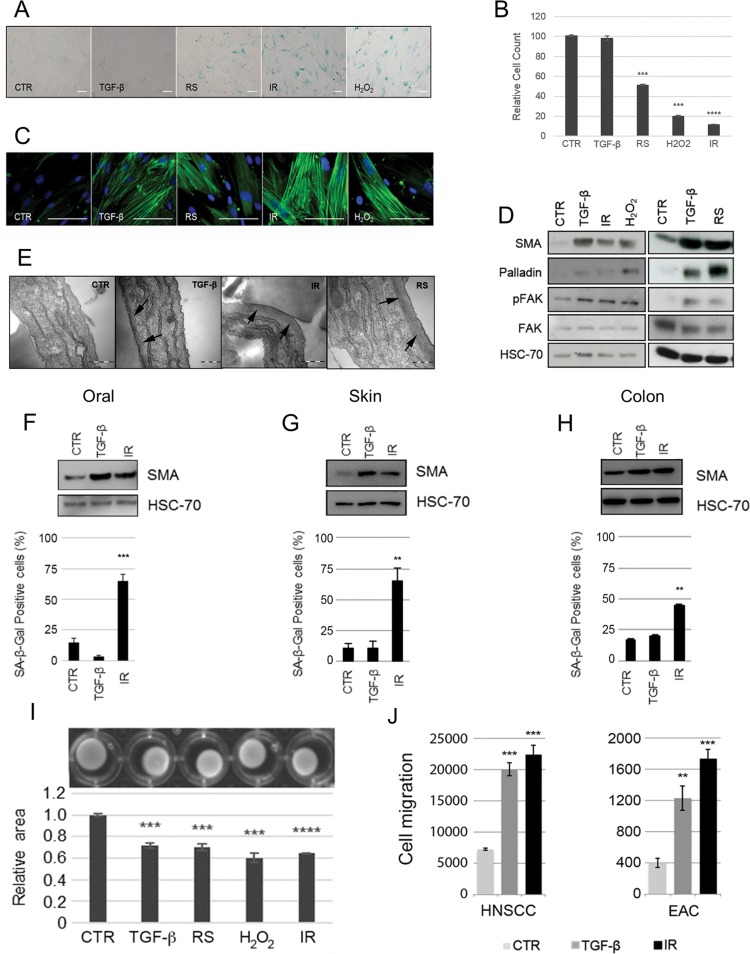
Induction of fibroblast senescence generates a myofibroblastic phenotype HFFF2 fibroblasts were induced to senesce through serial passaging (RS), treatment with γ-irradiation (10Gy; IR) or H_2_O_2_ (1mM). Cells were treated with TGF-β1 (2ng/ml) for 72 hours to induce myofibroblast transdifferentiation as a positive control. 4-6 days post stimuli, induction of senescence was confirmed by (**A**) expression of SA-β-Galactosidase (SA-β-Gal; Scale Bar indicates 100μm) and (**B**) proliferation assays (cell counts presented as percentage of cells compared to untreated control cells; see also Supplementary Fig. S2A). Cells were examined for myofibroblast features: (**C**) Representative images of immunofluorescence for SMA expression (green) with DAPI nuclear counterstain (Blue) (Scale Bar indicates 100μm); (**D**) Western blotting for SMA, palladin and pFAK (HSC-70 as loading control); (**E**) Representative images of transmission electron microscopy. Arrows highlight sub-membranous microfilaments (Scale Bar indicates 50nm). (**F-H**) Western blotting for SMA expression (HSC-70 as loading control) and SA-β-Gal quantification following TGF-β1- and irradiation of primary fibroblasts isolated from oral, skin and colonic mucosa respectively. (**I**) Representative images of collagen gel contraction assays following treatment of HFFF2 with TGF-β1 or different senescence-inducing stimuli. Histogram shows quantification of gel area expressed as the mean ± SEM of 4 replicates; (**J**) Transwell migration assays with HNSCC (5PT) and EAC (OE33) cell lines. Conditioned media (CM) from HFFF2 fibroblasts induced to senesce through γ-irradiation (10Gy; IR) or to transdifferentiate into myofibroblasts through TGF-β1 was used a chemoattractant in the lower chamber. Data are presented as mean ± SEM and statistics are shown for T-test compared to controls (*p<0.05, **p<0.01, ***p<0.001, ****p<0.0001). See also Supplementary Fig. S1.

The high proportion of SMA-positivity in the senescent fibroblast population *in vivo* raised the possibility that the induction of senescence may promote myofibroblast differentiation. To investigate this *in vitro* we used human fetal foreskin fibroblasts (HFFF2), induced to senesce using different stimuli (irradiation, H_2_O_2_, replicative senescence, cisplatin) or treated with TGF-β1, to promote myofibroblast phenotype. Senescence was confirmed by increased expression of senescence markers SA-β-Galactosidase (SA-β-Gal; Fig. 2A), pH_2_AX and p21 (Supplementary Fig. S2A) and decreased proliferation rates (Fig. 2B). Myofibroblast transdifferentiation was confirmed by SMA-positive stress fiber formation (Fig. 2C), and upregulation of SMA, palladin and phospho-FAK (Fig. 2D). Notably we observed that, similar to TGF-β1 treatment, fibroblasts induced to senesce by different stimuli formed SMA-positive stress fibers (Fig. 2C) and showed increased expression of classical myofibroblast markers (Fig. 2D). SMA co-localized with SA-β-gal expression in senescent cells (Supplementary Fig. S2B), correlated with senescence in a dose-dependent manner following H_2_O_2_ treatment (Supplementary Fig. S2C) and was impaired by inhibition of the DNA-damage response pathway via a specific inhibitor of ATM (Supplementary Fig. S2D). Ultrastructural analysis confirmed that senescent fibroblasts contained abundant rough endoplasmic reticulum and bundles of submembranous microfilaments consistent with a myo-fibroblastic phenotype (Fig. 2E). These observations were reproduced in primary fibroblasts isolated from normal oral mucosa, skin and colon (Fig. 2F-H), and also using cisplatin as a further stimulus of senescence (Supplementary Fig. S2E, F). Similar to TGF-β1-treated HFFF2, SMA-positive cells generated through senescence were contractile (Fig. 2H) and supported Transwell migration of tumor cell lines from cancers of the head and neck (HNSCC, 5PT) and esophagus (EAC, OE33) (Fig. 2I). These data show that senescent fibroblasts develop a contractile myofibroblast-like phenotype similar to TGF-β1 treatment, which is consistently observed across multiple senescence stimuli and in primary fibroblasts isolated from different tissues.

### Senescence stimuli induce a myofibroblastic phenotype through activation of SMAD signaling

**Figure 3:**
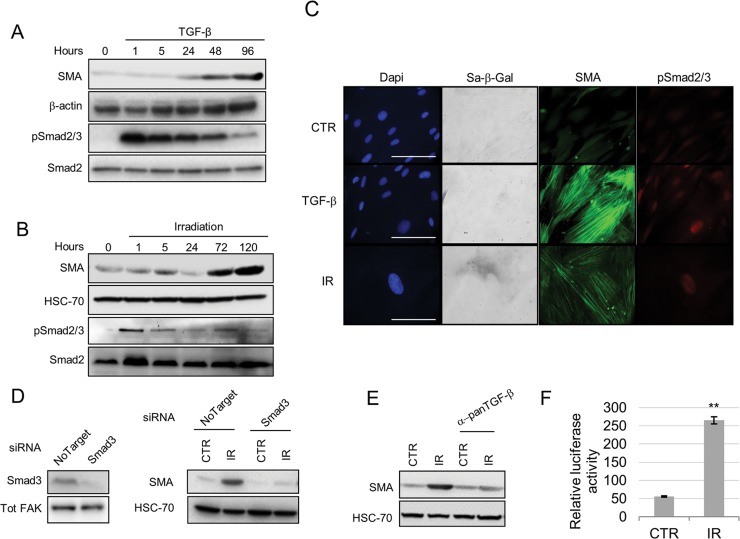
Smad signaling mediates senescence induction of a myofibroblastic phenotype Western blot showing SMA and phospho(p)-Smad2/3 expression following treatment of HFFF2 fibroblasts with TGF-β1 (**A**) or irradiation (**B**) (Smad2 and HSC-70 as loading controls). (**C**) Representative images of immunofluorescence on HFFF2 fibroblasts showing expression of SMA (green), pSmad2/3 (red), SA-β-Galactosidase activity (grey; in bright field) with DAPI nuclear counterstain (Blue) (scale bar indicates 100μm). (**D**) Western blots showing SMAD3 knock-down (Tot-FAK as loading control) (left) and SMA expression (HSC-70 as loading control; right) in irradiated HFFF2 cells. (**E**) Western blot for SMA expression in irradiated HFFF2 pre-treated with a pan TGF-β1 inhibitory antibody. (**F**) TGFβ-1 assay showing luciferase activity controlled by a TGFβ-1 responsive promoter in MLEC cells co-cultured for 24 hours with untreated or irradiated fibroblasts (y axis indicates the ratio between luciferase activity and HFFF2s protein concentration). Data are presented as mean ± SEM and statistics are shown for T-test compared to controls. See also Supplementary Fig. S3.

Canonical TGF-β/Smad signaling plays a pivotal role in myofibroblast differentiation [6] and has also been shown to play a role in senescence induction [22]. To investigate the molecular mechanism regulating senescence-induced myofibroblast differentiation we examined Smad activation following senescence induction through irradiation. Similar to TGF-β1 treatment, irradiation increased phospho-Smad2/3 protein levels and its nuclear localization (Fig. 3A-C). Other senescence-inducing stimuli (replicative senescence, H_2_O_2_) similarly activated Smad signaling (Supplementary Fig. S3A). Inhibiting TGF-β signaling using siRNA knockdown of Smad3, a pan-TGF-β antibody or a specific inhibitor of TGF-β receptor 1 (TGF-βR1) kinase activity suppressed SMA upregulation by irradiation and H_2_O_2_ (Fig. 3D, E; Supplementary Fig. S3B-D). We also found that irradiated fibroblasts have enhanced ability to activate TGF-β1, which may play an important role in driving myofibroblastic transdifferentiation in adjacent cells (Fig. 3F). These data show that induction of senescence activates canonical TGF-β1 signaling which promotes myofibroblast transdifferentiation.

### Transcriptomic analysis of myofibroblasts induced through TGF-β1 or senescence

Due to their functional and molecular similarities, we next compared the transcriptomic profiles of HFFF2 fibroblasts treated with either TGF-β1 or irradiation, cultured for 7 days and analyzed by RNA-sequencing.

**Figure 4:**
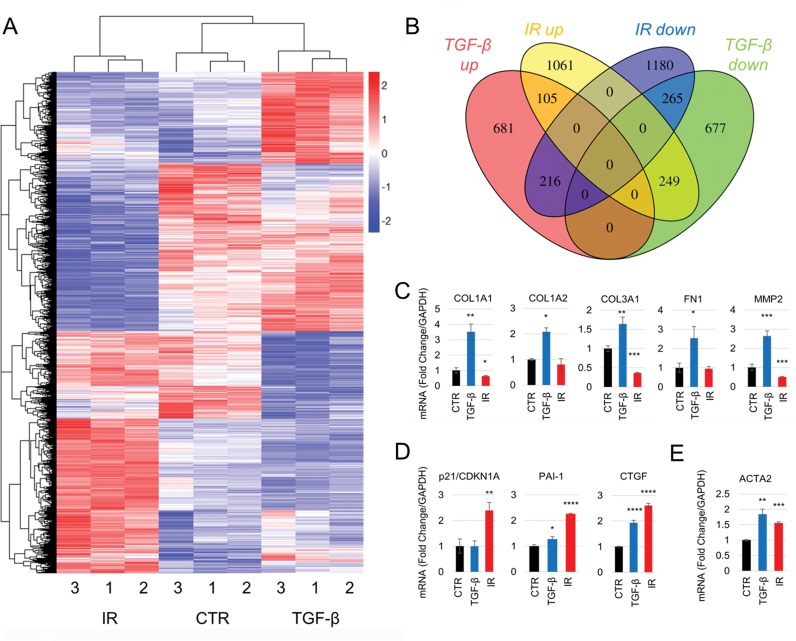
Senescence- and TGF-β1-induced myofibroblasts have divergent gene expression profiles (**A-B**) RNA-sequencing analysis of HFFF2 cells treated with TGF-β1 (2 ng/ml) or irradiation (10Gy) and grown for 7 days. (**A**) Unsupervised hierarchical clustering of the expression levels of differentially expressed genes (DEGs; FDR adj. p<0.001), identified using GLM likelihood ratio testing. Expression levels were subjected to Z score scaling within each sample for visualization purposes. Distances were calculated using a Euclidean distance measure. (**B**) Venn diagram showing the number of DEGs up- or downregulated in TGF-β1 and irradiated fibroblasts compared to controls. (**C-E**) RT-PCR measurements of mRNA expression levels of genes associated with myofibroblasts (**C, E**) and senescence (**D**) in HFFF2 cells used in the RNA-sequencing. Data are presented as mean ±SEM and statistics are shown for t-tests compared to control group. See also Supplementary Fig. S4.

Gene Set Enrichment Analysis confirmed that the expression profiles of the treatments matched TGF-β1 and senescent gene signatures described previously ([29, 30] respectively; Supplementary Fig. S4A-B). Given the functional and molecular similarities identified in the earlier experiments, we were surprised that unsupervised hierarchical clustering of significant differentially expressed genes (DEGs; FDR adj. p<0.001; Supplementary File 1) clearly discriminated between the subgroups; this highlighted a relatively small number of genes commonly up-regulated (5.7%) or downregulated (12.5%) (Fig. 4A, B), including *ACTA2* (SMA). We confirmed the RNA-sequencing results using real-time PCR, focusing on genes known to be up-regulated in myofibroblasts and senescent fibroblasts. Notably, genes associated with ECM deposition and manipulation (*COL1A1/1A2/3A1*, *FN1* and *MMP2*)[31] (Fig. 4C), were only significantly upregulated in TGF-β1-induced myofibroblasts. Conversely, senescence markers (*CDKN1A/p21* and *SERPINE1/PAI-1*) [32] were primarily up-regulated in irradiated cells (Fig. 4D). We confirmed that *ACTA2* was up-regulated in both subgroups (Fig. 4E).

We used Gene Set Enrichment Analysis to determine the gene ontology terms associated with the TGF-β1 and irradiated transcriptomes. This showed that TGF-β1-treated fibroblasts up-regulate a number of genes associated with ECM deposition/manipulation, muscle development and proliferation, consistent with a classical myofibroblast phenotype. Comparatively, irradiated fibroblasts up-regulated genes associated with contractility, but no GO terms related to ECM deposition/manipulation were identified (Table 2 and Supplementary Table S2 for the full list of GO categories). These data show that while TGF-β1-driven myofibroblasts and senescent fibroblasts share common genes associated with cell contractility, the phenotype associated with ECM production/remodeling is specific to the TGF-β1-treated cells.

**Table 2:** Top 5 GO terms with significant enrichment of TGF-β1-up or IR-up DEGs, identified by Gene Set Enrichment Analysis

**TGF-β1 Up-regulated**				
**GO TERM**	**SIZE**	**NES**	**NOM p**	**FDR q**
PROTEINACEOUS EXTRACELLULAR MATRIX	72	2.001	<0.001	0.067
EXTRACELLULAR MATRIX	73	1.998	<0.001	0.035
SKELETAL DEVELOPMENT	71	1.902	<0.001	0.094
INTERPHASE OF MITOTIC CELL CYCLE	57	1.846	<0.001	0.140
DNA REPLICATION	95	1.817	<0.001	0.159
				
**Irradiation Up-regulated**				
**GO TERM**	**SIZE**	**NES**	**NOM p**	**FDR q**
CONTRACTILE FIBRE PART	15	2.149	<0.001	0.013
CONTRACTILE FIBRE	16	2.067	<0.001	0.021
REGULATION OF PROTEIN AMINO ACID PHOSPHORYLATION	18	1.893	<0.001	0.117
EXTRINSIC TO MEMBRANE	15	1.870	<0.001	0.111
STRUCTURAL CONSTITUENT OF MUSCLE	18	1.821	0.013	0.161

### Senescence-induced myofibroblasts develop a non-fibrogenic phenotype *in vitro* and *in vivo*

**Figure 5:**
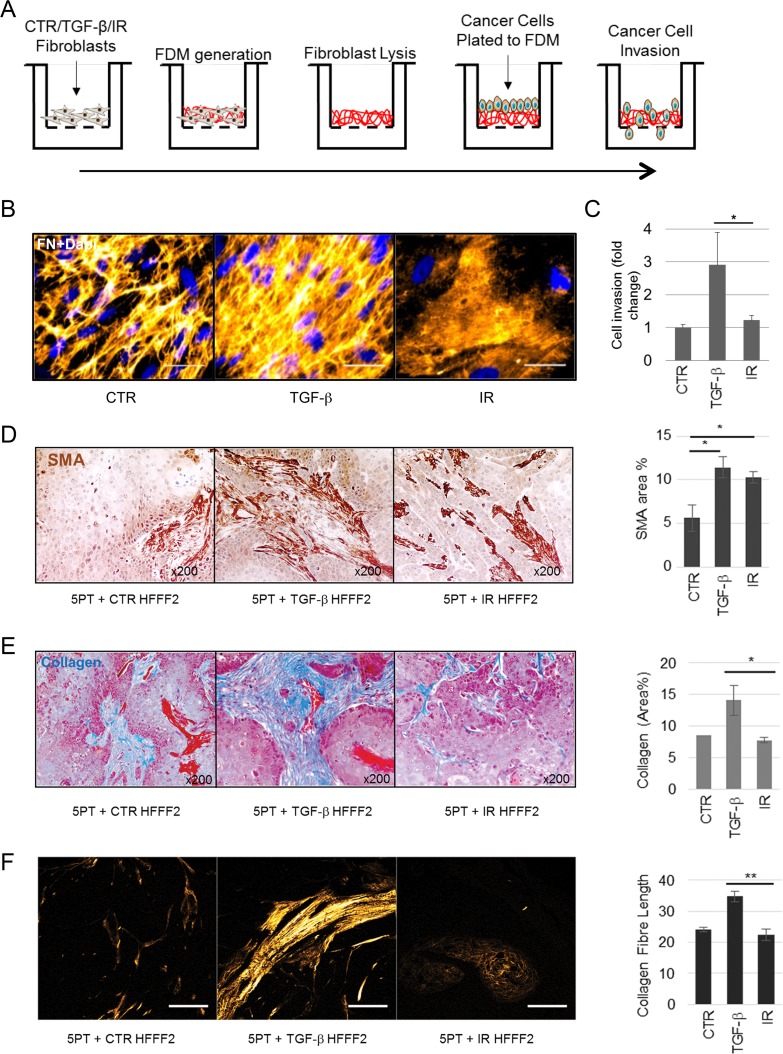
Myofibroblasts and not senescent fibroblasts mediate collagenous ECM deposition (**A**) Schematic of experimental procedure for B and C. (**B**) Representative image of immunofluorescent staining for Fibronectin (FN) in fibroblast-derived matrices (FDM) produced by HFFF2s treated as indicated; FN (orange; pseudo-colored in Fiji) and Dapi (blue) as nuclear counterstain; scale bar represents 50μm. (**C**) Transwell assay examining OE33 invasion through FDM deposited by HFFF2 fibroblasts induced to transdifferentiate through treatment with TGF-β1 or γ-irradiation (IR). (**D-F**) Analysis of xenografts formed from 5PT cells injected s.c. into RAG1^−/−^ mice with HFFF2 fibroblasts treated as indicated. (**D**) Representative images of SMA immunochemistry in 5PT xenografts co-injected with HFFF2s treated as indicated. Histogram shows SMA quantification expressed as % positive area. (**E**) Representative images of Masson's trichrome staining for collagen (royal blue) with HFFF2s treated as indicated. Histogram shows quantification expressed as % positive area. (**F**) Representative images showing multi-photon excitation (MPE) filtered for second harmonic generation to identify collagen fibers on sections from the xenograft tumors as indicated (Scale Bar indicates 100μm). Histogram shows quantification of collagen fiber elongation. Data are presented as mean ± SEM and statistics are shown for T-test compared to controls unless otherwise indicated.

The difference in ECM related gene expression, between TGF-β1 treated and irradiated fibroblasts, was clearly demonstrated by analysis of genes within the core ‘Matrisome’ ([33]; consisting of fibronectin, proteoglycans and collagens, among other ECM associated genes). These genes were predominantly up-regulated in TGF-β1-treated fibroblasts and downregulated in irradiated fibroblasts (Supplementary Fig. S4C). Consistent with these findings, *in vitro* analysis of fibroblast-derived matrices (FDMs) showed that TGF-β1-treated fibroblasts produced a dense fibronectin matrix composed of mature fibrils; whereas irradiated fibroblasts produced minimal matrix (Fig. 5A-B). In contrast to Transwell assays using fibroblast conditioned medium (showing that both TGF-β1 and irradiated myofibroblasts secrete factors that promote tumor cell migration; Fig. 2J), tumor cells showed significantly increased invasion only through matrices secreted by TGF-β1-treated fibroblasts (Fig. 5C), suggesting that this ECM differentially promotes cancer cell invasion.

As collagens are a major constituent of tumor ECM, we next interrogated the RNA-sequencing dataset to examine expression of collagen superfamily genes (Fig. 5B-C). These genes were predominantly upregulated by TGF-β1 and down-regulated following irradiation (Supplementary Fig. S4D; complete lists are provided in Supplementary Table S3). Notably, there was complete divergence in the expression of fibrillar collagens (each being up-regulated by TGF-β1 and down-regulated by irradiated fibroblasts) (Fig. S4E). This was also confirmed at the protein level by Western blotting and immunofluorescence (Supplementary Fig. S4F and G).

To test the *in vivo* ability of SMA-positive fibroblasts, generated either through TGF-β1 treatment or senescence induction, to produce and remodel collagenous ECM, we used a HNSCC cancer xenograft model (5PT) [34], co-injecting tumor cells with TGF-β1-treated or irradiated fibroblasts. Consistent with our earlier observations, both fibroblast subgroups generated an SMA-positive tumor stroma (Fig. 5D). However the analysis of collagen deposition and fiber organization by Masson's Trichrome staining and Second Harmonic Generation imaging on the mouse tumors showed that TGF-β1 treated fibroblasts generated significantly more collagen with elongated fibers compared to irradiated fibroblasts (Fig. 5E-F). This evidence suggests that although senescent fibroblasts display a SMA-positive contractile phenotype they have reduced ability to deposit collagenous ECM.

### Analysis of ECM in human tumors in relation to myofibroblast subsets, and association with patient survival

**Figure 6:**
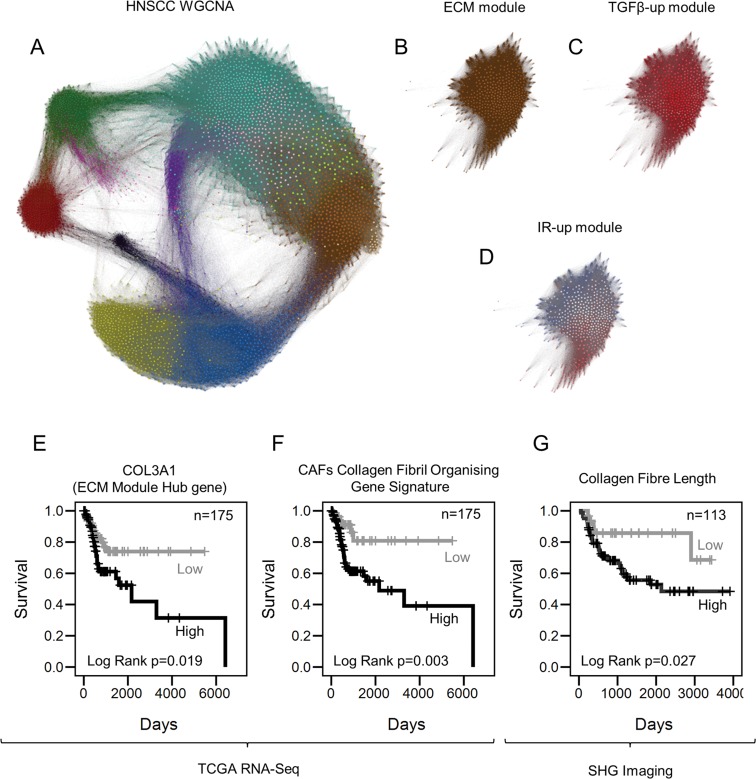
Collagen fiber deposition impacts tumor progression (**A**) Gephi network graph where each node represents a gene labelled by color according WGCNA module assignment. Distance between nodes is represented by the TOM connectivity measure. The brown module is the ECM module. (**B**) ECM module extracted from panel A. (**C**) Network graph of the ECM module where nodes are color coded according to the correlation with TGF-β1-up DEGs, summarized by a signature eigengene. Red and blue colors show positive and negative correlation, respectively. (**D**) Correlation of IR-up DEGs with members of the ECM module (color range described above). (**E-F**) Kaplan-Meier curves showing survival rates in HNSCC patients with greater than average expression of genes associated with myofibroblasts stratified for COL3A1 (**E**) and CFOG expression (**F**). (**G**) Kaplan-Meier curves showing disease specific survival (DSS) rates in HNSCC patients with moderate or high stromal SMA expression (measured by immunohistochemistry), stratified by collagen fiber elongation measured by Second Harmonic Generation imaging. See also Supplementary Fig. S5.

To determine the association between senescent-myofibroblasts or TGF-β-myofibroblasts and gene expression in human tumors we applied weighted gene co-expression network analysis (WGCNA) to the Cancer Genome Atlas HNSCC RNA-sequencing dataset (TCGA Network, 2015) [35]. This systems analysis of gene co-expression allowed us to extract information on cell-specific biological processes occurring in whole tissue of 246 HNSCC cases identifying twelve modules of highly co-expressed genes (Fig. 6A) [36]. These included an ECM module significantly enriched with collagen superfamily genes (brown color, *ECM module;* GO: ECMO; see Supplementary File 2 for the GO analysis; Fig. 6A-B) [36]. The *ECM module* correlated well with TGF-β1-up DEGs derived from our *in vitro* RNA-sequencing analysis (KME=0.94, p≤2.2E^−16^) and with a publicly available TGF-β1 geneset used in Supplementary Fig. S4A (KME=0.529, p<2.2E^−16^) (Fig. 6C and Supplementary Fig. S5A, respectively). However, there was no or weak correlation between the ECM module and irradiation-up DEGs (KME=-0.022, p=0.631) or with the senescence geneset used in Supplementary Fig. S4B (KME=0.103, p=0.023) (Fig. 6D and Supplementary Fig. S5B). Consistent with this, collagen superfamily genes showed a strong positive correlation with the TGF-β1 gene signature, but not the irradiation-gene signature (e.g. COL1A1-TGF-β1 r=0.92 and COL1A1-irradiation r=0.23; Supplementary Fig. S5C and Supplementary Table S4). Analysis of fibrillar collagen structure in human HNSCC stroma confirmed the correlation observed in the bioinformatics analysis between ECM production and a myofibroblastic non-senescent stroma: p16-negative/SMA-positive tumors displayed elongated collagen fibers which were absent in the p16-positive/SMA-positive stroma (Supplementary Fig. S5D-F). Overall, these data suggest that ECM production and organization are restricted to CAFs with non-senescent myofibroblastic phenotype and not found in senescent CAFs.

We have shown previously that a myofibroblastic SMA-positive stroma is associated with poor survival in HNSCC and EAC [8, 10], but the results above describe heterogeneity within this population, particularly in the ability to produce collagenous ECM. We tested whether this related to patient survival rates using the HNSCC CGA cohort. The cohort was divided into two based on the mean expression of genes associated with myofibroblasts (ACTA2, CDH2, PDGFRA, PDGFRB, VIM, S100A4) to examine the prognostic significance of the ECM module hub gene COL3A1 (the most connected gene, representative of the ECM module expression) within HNSCC cases with a myofibroblastic stroma; this analysis showed COL3A1 expression is associated with poor survival (Log Rank p=0.019; Fig. 6E). We previously identified a cluster of collagen fibril organizing genes (CFOG: COL1A1, COL1A2, COL3A1, P4HA1, LOX, LEPRE1, ANXA2, TNXB, PLOD3, SERPINH1) up-regulated in a fibrogenic sub-set of SMA-positive CAFs [14]. High expression of this gene signature was also associated with poor prognosis in the SMA-positive HNSCC patient group (Log Rank p=0.003; Fig. 6F).

Finally we used Second Harmonic Generation imaging to analyze fibrillar collagen in the tumor stroma of HNSCC and EAC patient cohorts selected for moderate/high stromal SMA expression [14]. This showed that the presence of elongated collagen fibers was significantly prognostic for both tumor types (Log Rank p=0.027 & 0.028 respectively; Fig. 6G, Supplementary Fig. 5G & Supplementary Tables S5 and S6). These data indicate that, within the myofibroblastic CAF population, the ‘classical’ matrix-producing phenotype is associated with the poorest survival.

## DISCUSSION

While numerous markers, such as PDGFRα, periostin and fibroblast activation protein (FAP) have been reported to identify CAF within the tumor microenvironment [37], SMA is most commonly used to identify those CAF with an ‘activated’ tumor-promoting myofibroblastic phenotype. The importance of SMA-positive CAF in tumor progression is highlighted by the poor survival rates of patients with this type of tumor stroma, with contractile cells likely to actively contribute to tumor progression through generating increased tissue tension and promoting tumor cell mechanotransduction, invasion and metastasis [11-13, 38, 39]. Indeed, the degree of *in vitro* myofibroblast contractility has been shown to correlate with tumor promotion *in vivo* [2], and studies using 3D model systems have shown that mechanical re-modelling of the ECM through cellular contraction is a requirement for cancer cell invasion [8, 39].

Similar to myofibroblastic CAF, senescent CAF have also been shown to be tumor promoting in model systems [19-21]; although few studies have examined their frequency, prognostic significance or relationship to myofibroblastic CAF in human tumors [40]. Senescence has been shown to limit the progression of myofibroblast-dependent tissue fibrosis [24-26], and suggestions have been made that myofibroblast differentiation and senescence reflect a unified program of fibroblast differentiation. Consistent with this, we found that senescent CAF in different tumor types are mostly SMA-positive. Although CAFs are sometimes considered to be highly proliferative cells, we found that CAF isolated from HNSCC, and cultured *ex vivo*, show senescent features, which correlate with SMA positivity [41]. Notably, here we show that induction of fibroblast senescence generates an SMA-positive contractile cell with ultrastructural myofibroblastic features. However, despite TGF-β1-induced and senescence-induced myofibroblasts having similar contractile and functional features, there are major differences in their gene expression profiles, clearly observed in the expression of genes associated with ECM deposition and organization, suggesting that these cells generate significantly different microenvironments during tumor progression. Proteomic analysis of SMA-positive CAF isolated from EAC has previously highlighted variations in collagen gene expression [14]; our current study suggests that this may be due to a variably senescent phenotype.

Transwell experiments show that both types of SMA-positive CAF secrete factors that promote tumor cell motility, although these are mostly different. For example, analysis of RNA sequencing data from SMA-positive cells generated through either TGF-β1 treatment or senescence-induction revealed contrasting upregulation of numerous matrix and soluble factors reported previously to promote tumor invasion (eg. TGF-β1 treatment - *IGF1, PDGF-A, VEGF-A, MMP-2, POSTN* (periostin); senescence induction – *SPP1* (Osteopontin), *CXCL14, FGF1, EGF, MMP-12*; all p<0.001); we recently found that suppression of the SASP in senescent fibroblasts through mTOR inhibition abrogates this invasion-promoting effect [42].

Induction of SMA expression and cell contractility following various senescence stimuli was dependent on TGF-β/Smad signaling. This is consistent with previous studies indicating that radiation and reactive oxygen species can activate latent TGF-β1 [43]. Conversely we, and others, have also observed that TGF-β/Smad pathway is crucial for induction of senescence [41, 44, 45], suggesting a degree of overlap between the molecular mechanism(s) regulating myofibroblast differentiation and senescence. RNA-sequencing data showed that senescent fibroblasts are characterized by reduced ECM deposition, and previous studies on liver fibrosis and skin wound healing have indicated that myofibroblasts, whilst initially proliferating and producing ECM, are themselves eventually driven into senescence and cleared by the immune system, thereby self-limiting fibrogenesis [24, 25]. The matricellular protein CCN1 has been shown to restrict fibrosis in senescent fibroblasts [25]; consistent with this, our RNA sequencing analysis revealed CCN1 upregulation in senescent, SMA-positive myofibroblasts generated through irradiation (p=0.003). Thus myofibroblast differentiation and fibroblast senescence may form part of a dynamic spectrum of fibroblast responses found at different stages along the same regulatory program, and it is possible that the pathological accumulation of SMA-positive, ECM-producing myofibroblasts in cancers and progressive fibrosis may result from an ongoing process of senescence evasion or continuous myofibroblast repopulation.

Survival analysis of cohorts of patients with HNSCC and EAC showed that collagen deposition and structure is significantly prognostic, and adds a further layer of stratification in patients with tumors containing an SMA-positive stroma. While most studies have shown that senescent fibroblasts are generally tumor promoting, this being linked to secreted factors within the SASP, within the SMA-positive CAF population they can be regarded as the lesser of two evils. It remains to be addressed whether the difference in prognosis between the SMA-positive CAF subgroups is due to deposition/reorganization of collagenous matrix; it is possible that the presence of a collagen-rich ECM is simply a surrogate for identifying the presence of cells with a ‘conventional’ myofibroblast phenotype. Further studies are required to examine whether the ECM remodeling capability or the secretory program of myofibroblastic CAFs has the strongest impact on tumor progression. However, our Transwell assays show that while TGF-β-driven myofibroblasts promote tumor cell motility by secreting factors and manipulating ECM, senescent fibroblasts lack the latter property. Several studies suggest mechanisms through which collagen may promote cancer progression; generating a stiff ECM that activates integrin-dependent mechanotransduction pathways and promotes cell motility [38]; providing aligned tracks that guide and facilitate cancer cell movement and dissemination [46]. Consistent with these reports, collagen structure has been shown to be prognostic in breast cancer [47, 48] and increased expression of type I collagen and related genes is frequently observed in the gene expression signatures associated with increased risk of metastasis [49].

In summary, these data describe a common link between myofibroblast differentiation and senescence. Senescent fibroblasts represent a distinct subgroup of cells within the SMA-positive, myofibroblastic CAF population, sharing a contractile phenotype but not the ability to generate an organized collagenous ECM. This latter feature predicts for outcome in cancer patients, improving prognostic stratification and refining characterization of the activated ‘myofibroblastic’ phenotype.

## MATERIALS AND METHODS

### Analysis of human tumors

Appropriate ethical and institutional approval to work with human tissues was obtained. Tissue microarrays of head & neck cancers (HNSCC) [8] and esophageal adenocarcinoma (EAC) [10] were constructed from archival paraffin-embedded material at University Hospital Southampton using triplicate, randomly-selected 1mm cores (Alphelys MiniCore 3). Scoring for SMA and p16 staining was carried out independently (GJT, KM, TU; blinded to clinical outcome) using a semi-quantitative scoring system [8], according to the extent of stromal positivity (low/negative [<5% stroma positive], moderate [patchy/focal expression, 5–50% stroma positive], high [diffuse expression throughout tumor, >50% stroma positive]). Immunohistochemistry methodology is described in online Supplementary Materials.

### Cell Culture

Human EAC cell line OE33, and human fetal foreskin fibroblasts HFFF2 were purchased from European Collection of Cell Cultures (Public Health England); HNSCC 5PT [34] was provided by I. Mackenzie (Queen Mary University of London). Mink lung epithelial cells (MLEC) stably expressing a TGF-β1-responsive luciferase reporter construct were provided by D. Rifkin (New York University); HFFF2 cells, MLEC and human primary fibroblasts isolated *ex vivo* were cultured in Dulbecco's modified Eagle's medium (DMEM) supplemented with 10% FBS and 292μg/ml L-Glutamine. OE33 were cultured in RPMI-1640 medium supplemented with 10% FBS and 292μg/ml L-Glutamine. 5PT were cultured in keratinocyte growth medium [50]. HFFF2 and human primary fibroblasts were used at early passage (1-10; see online Supplementary Material for primary fibroblasts isolation). All cells were cultured using standard polystyrene cell culture plates/flasks (Corning). HFFF2 cells were transfected with SMAD3 On-Target pool siRNA (Thermo Scientific/ Dharmacon) using Oligofectamine reagent (Life Technologies) as described [51]. TGF-β1 (R&D Systems; 2ng/ml for 3 days) was used to induce myofibroblast differentiation unless otherwise stated. TGF-βR1 inhibitor (1μM; Calbiochem), TGF-β1-2-3 antibody (10μg/ml; MAB1835, R&D Systems) or ATM inhibitor KU-55933 (20μM; Millipore) were added to cells 2-hours prior to TGF-β1 treatment. Conditioned medium (CM) was prepared over 72 hours as described previously [50] from cells treated with TGF-β1 or with senescence-inducing stimuli, and used as a chemoattractant in Transwell migration assays [52] (online Supplementary Materials).

### Senescence induction

To induce replicative senescence, fibroblasts were serially passaged (to passage 50) and senescence confirmed as described below. To induce pre-replicative senescence confluent pre-senescent fibroblasts were detached and irradiated in suspension with 10Gy γ-rays (1-2 cycles of 10Gy) and plated at sub-confluent densities [53]; otherwise adherent, fibroblasts were treated with either 1mM H_2_O_2_ or 10μM Cisplatin, and re-plated after 24 hours at sub-confluent densities. Cells acquired a fully senescent phenotype 4-6 days post-treatment, confirmed by determining the percentage of SA-β-Gal-positive cells (Sigma) with cells counterstained with DAPI [54]. At least 200 cells/well in duplicate were counted per sample.

### Second Harmonic Generation Microscopy

Fibrillar collagen was imaged using a custom-built two-photon laser scanning microscope at the Laboratory for Optical and Computational Instrumentation (LOCI) University of Wisconsin-Madison; and quantitatively analyzed using the ctFIRE software package (http://loci.wisc.edu/software/ctfire) [48, 55]. Measu-rement of collagen fiber elongation is described in online Supplementary Material.

### *In vivo* experiments

Investigation on mice has been conducted in accordance with the ethical standards and according to the Declaration of Helsinki and according to national and international guidelines and has been approved by the authors' institutional review board and by the Home Office. Xenograft model: 1×10^6^ 5PT cells [34] ± 3×10^6^ HFFF2 cells were re-suspended in 150μl of supplement free DMEM; 100μl of this mix was injected s.c. in the flank of partially immunocompromised, male, RAG1−/− C57BL/6 mice. Animals were culled after 5-6 weeks of tumor growth and tumors processed to paraffin. At least 5 animals were used per group. Quantification of SMA and Masson's Trichrome (collagen) staining in xenograft tumors was carried out using color thresholding in Fiji. 3-5 independent, randomly selected, fields of view were analyzed for each tumor in order to generate a mean value per tumor, which was compared between treatment groups.

### RNA-sequencing

HFFF2 were treated 7 days prior to RNA extraction, RT-PCR and RNA-sequencing analysis. RNA-sequencing libraries were prepared for each RNA sample, sequenced using an Illumina HiSeq 2500 platform (see online Supplementary Materials). FDR adj. p<0.001 was used to determine expression levels of differentially expressed genes (DEGs) (see Supplementary Material for full list of DEGs). Gene Set Enrichment Analysis was performed using the Broad Institutes GSEA tool with gene lists pre-ranked by log fold-change (http://www.broadinstitute.org/gsea/).

### Bioinformatic analysis

#### Data acquisition and processing

CEL files were imported from the HNSCC dataset from Cancer Genome Atlas Network [35] into the R/Bioconductor package ‘limma’ and normalized using the voom algorithm [56, 57]. Outlier subjects were identified and excluded using a sample network connectivity statistic described previously [58] and implemented in the R function SampleNetwork.

#### Weighted Gene Co-expression Analysis (WGCNA)

WGCNA is a systems data mining method used for studying gene co-expression networks based on pairwise correlations between variables., and was performed using the WGCNA package in R[58]. This was used to extract information on cell-specific biological processes occurring in whole tumor tissue of 246 HNSCC cases (Cancer Genome Atlas Network) [35]. The power of WGCNA in capturing a systems perspective is built upon its underlying algorithm, which takes into account not only the correlation of two genes with each other but also the degree of similarity between a pair of genes in their correlation structure within the rest of the network [36]. Briefly, a signed correlation matrix was obtained by calculating the weight mid-correlations between all variable probe sets across all samples. Next, the adjacency matrix was calculated by raising the absolute values of the correlation matrix to a power of 12. For computational reasons, topological overlap (TO) was then calculated for the 4000 most connected genes (among the 9925 most varying annotated probe sets) across 246 samples. Finally, genes were hierarchically clustered using 1-TO (topological overlap) as the distance measure and twelve differently colored modules were determined using a dynamic tree-cutting algorithm. To establish whether the gene co-expression modules reflect cell types or biological processes, a Gene Ontology (GO) analysis was conducted using the ToppGene bioinformatics suite [59]. To determine if the network was reproducible across datasets, we evaluated that the module density and connectivity patterns defined in the CGA RNA-sequencing dataset were preserved in two independent HNSCC cohorts [60, 61] both analyzed with Affymetrix HG-U133 Plus 2.0 microarrays. 88.9% of the 4000 genes used to construct the aforementioned network were preserved in the array data. The relative affiliation (Membership) of any gene or gene group to the ECM module was estimated by correlating its expression profile with the first principal component of the module, termed the module eigengene (ME)[58]. KME are Module Membership values, also known as eigengene-based connectivity, and range from 0 (indicating no connectivity) to +1 (high connectivity) or to −1 (inverse connectivity). The network was graphically depicted by exporting the TO weights into the program Gephi [62].

### Statistical analysis

Statistical analysis of patient survival rates was performed using SPSS v22 (IBM SPSS Inc.). The primary endpoint was death from cancer; survival time was measured from the date of diagnosis in the HNSCC cohort and date of surgery in the EAC cohort. Other causes of death were censored at the time of death. Kaplan-Meier plots (with Log-rank [Mantel-Cox] tests) were used to describe the risk of dying from cancer within the indicated stratification metrics, unless otherwise stated.

Throughout the manuscript, all the experiments were performed at least 3 times and data are expressed as the mean +/− SEM of at least 3 replicates. Where appropriate, one way analysis of variance (ANOVA) was used to compare multiple groups. For comparisons between groups a two-tailed homoscedastic Student's t-test was used. A *p* value of < 0.05 was considered significant (*p<0.05, **p<0.01, ***p<0.001, ****p<0.0001).

Immunocytochemistry, Western blotting, Real Time Quantitative PCR (primer sequences described in Supplementary Table S7), generation of Fibroblast Derived Matrices (FDMs), Transmission Electron Microscopy, collagen gel contraction, migration and TGF-β1 assays are described in the Supplementary Materials.

## SUPPLEMENTAL MATERIALS TABLES AND fIGURES








