# Supplementary material for: Induction of fibroblast senescence generates a non-fibrogenic myofibroblast phenotype that differentially impacts on cancer prognosis
Source: Aging (Albany NY). 2016 Dec 15;9(1):114–31. doi: 10.18632/aging.101127 (PMC5310659; doi:10.18632/aging.101127)
Supplement: Supplementary file 1 [file aging-09-0114-s001.pdf]

## SUPPLEMENTARY MATERIALS

Please browse the links in the **Full Text** version of this manuscript to see Supplementary Methods, Figures and Tables.

**Supplementary Materials.**

**Supplementary File 1.**

**Supplementary File 2.**
