# Supplementary material for: Induction of fibroblast senescence generates a non-fibrogenic myofibroblast phenotype that differentially impacts on cancer prognosis
Source: Aging (Albany NY). 2016 Dec 15;9(1):114–31. doi: 10.18632/aging.101127 (PMC5310659; doi:10.18632/aging.101127)
Supplement: Supplementary file 4 [file aging-09-0114-s004.pdf]

SUPPLEMENTARY MATERIALS

METHODS

Immunohistochemistry

Archival Samples & Human FFPE tissues

Automated immunostaining of full tissue sections or Tissue Microarrays was performed in a UKAS-accredited Cellular Pathology Laboratory in University Hospital Southampton. 4µm FFPE sections were mounted on Superfrost+ (Thermofisher scientific) slides and baked for 30 minutes at 65°C prior to use. All subsequent steps were completed using commercially available visualization systems [Envision FLEX (Dako)] and automated platforms [Dako PT Link (Dako); Autostainer Link48 (Dako)] optimized for use within a clinical diagnostic pathology laboratory.

Deparaffinisation, rehydration and antigen retrieval was completed for 20 minutes at 97°C using a predefined program on the Dako PT links. Antigenic retrieval varied depending on primary antibody: Envision FLEX High pH for SMA, p16, p21 and p53; Envision FLEX Low pH for 8OHDG and pH2Ax. Non-specific staining due to endogenous Peroxidases was blocked using Envision FLEX blocking reagent (Dako), prior to incubation with primary antibodies. Primary incubation times were selected based on previously established optimal conditions: 20 minutes for: SMA, p16, p21, p53 and pH2Ax; 30 minutes for: 8OHDG.

Secondary amplification and enzymatic conjugation was completed using Envision FLEX HRP (Dako, 20 mins) and either Rabbit Link (Dako, 15 mins; p16) or Mouse Link (Dako, 15 mins; p21) reagents as appropriate. Chromogenic visualization completed with 2x5 minute washes in DAB and counterstaining with Haematoxylin. Antibodies used were: SMA (M0851, 1:200, Dako), 8OHDG (Ab48508, 1:100, Abcam), p16 (Ab7962, 1:200, Abcam), p21 (M7202, 1:50, Dako), p53 (M7001, 1:30, Dako), pH2Ax (05-636, 1:100, Millipore). Images were taken using a Zeiss AX10 microscope.

FFPE Mouse Xenograft Tumors

Immunohistochemistry of mouse xenograft tumors was performed using the commercially available Mouse on Mouse immunohistochemistry detection kit (PK-2200, VectorLabs, Peterborough, UK) according to the recommended protocol. Quantification of SMA and Masson's Trichrome collagen staining was carried out using the 'Trainable Weka Segmentation' machine based learning plugin in Fiji [63].

Collagen staining

Collagen was stained using the Masson's Trichrome dye (Dako) on Artisan Link Pro automated platform (Dako) accordingly with manufacturer's instructions.

Fibroblasts isolation

Samples of normal human skin, colon or oral mucosa tissue were suspended in PBS supplemented with 100 units/ml penicillin, 100µg/ml streptomycin, and 0.25µg/ml amphotericin B. The specimen was cut into small pieces and placed into a 12-well plate, and cultured in DMEM supplemented with 20% fetal calf serum, 100u/ml penicillin, 100µg/ml streptomycin and 292µg/ml L-Glutamine. After 3-4 weeks fibroblasts were detached and characterized (cytokeratin-negative, CD31-negative, vimentin-positive) and then cultured in 10% FBS DMEM.

Fluorescent microscopy

Cells were plated for immunocytochemistry on permanox chamber slides (Thermo Scientific), fixed in 4% PFA. Immunostaining was performed as described previously [8]. DAPI or Topro3 were used as nuclear counterstains and coverslips were mounted using Fluorescent mounting solution (Dako). Images were taken using an Olympus IX81 fluorescence microscope and Xcellence program. For immunofluorescent staining of paraffin-embedded tissue, sections were dewaxed and rehydrated as described for immunohistochemistry, and then permeabilized in 0.2% Triton-X100, blocked with 2% BSA/0.02% Fish Skin Gelatin (Sigma)/10% FCS in PBS and incubated with primary antibody overnight. After three washes in PBS/0.1% Triton-X100, secondary antibody was applied and the experiment completed as previously described[8]. Antibodies used for immunofluorescence were: SMA (A5060, 1:750, Sigma), phosphoSMAD2/3 (3108, 1:100, Cell Signalling, Hitchin, UK), Fibronectin (F3648, 1:800, Sigma), Collagen V and p16 (ab189285, 1:200; ab7962, 1:100 respectively; Abcam).

Transmission Electron Microscopy

50 x 10^3 cells in 10% FBS DMEM were plated per Transwell for 3 days [Costar/Corning Transwell, 12 mm diameter, 0.4 um pore size polycarbonate membrane]. Cells were washed once with PBS and fixed overnight using 3% glutaraldehyde and 4% formaldehyde in 0.1 M PIPES buffer at pH 7.2 at 4°C. Transwells were washed (using 0.1 M PIPES buffer, pH 7.2) and incubated for 1 hour at room temperature with 1%

osmium tetroxide (in 0.1 M PIPES buffer at pH 7.2), washed and incubated for 20 mins in 2% aqueous uranyl acetate. Following dehydration through graded ethanol (from 30% up to 95% ethanol), the Transwell membrane was removed, incubated for 20 minutes in absolute ethanol (x2), followed by 10 mins in 100% acetonitrile at room temperature, and a mixture of 50% acetonitrile+50% Spurr resin at room temperature overnight. The acetonitrile/resin mixture was removed, and replaced with fresh resin at 60□C for 20-24 hours. Sections were cut using glass knives on a Reichert Ultracut ultramicrotome (OM U3) and digital images acquired on Hitachi H7000 transmission electron microscope.

Migration and proliferation assay

Transwell migration assays were performed as described previously [8, 50]. Briefly, cell migration assays were performed over 24h using uncoated polycarbonate filters (Costar). Cells migrating to the lower chamber were trypsinized and counted on a Casy 1 counter (Sharfe Systems GmbH). The Transwell invasion assay on Fibroblasts Derived Matrices was carried out using 10% FCS as a chemoattractant in the lower chamber of Transwells. Invading cells were quantified using crystal violet staining: cells in the upper chamber of the Transwell were removed using a cotton bud; the cells were then fixed in 4% Paraformaldehyde and stained using 0.1% crystal violet; the stain was then solubilized in 50% acetic acid and read at 595nm via spectrophotometry in a 96 well plate.
Proliferation assays were performed plating cells into 24-well plates, followed by trypsinization and counting on a Casy 1 counter.

Western blotting

Cells were lysed and sonicated in Laemmli buffer. Equal amounts of proteins were electrophoresed in 10-12% SDS-PAGE gels and electro blotted to PVDF membranes (Millipore) as previously [8]. Bound antibodies were detected using a chemiluminescence system (Pierce) and visualized at the Fluor-S Multi-imager (Bio-Rad). Antibodies used for Western blotting were: α-SMA, α-βActin (A2547, 1:1000, A5060, 1:1000 respectively, Sigma), HSC-70, p21 (Sc-7298, 1:1000, sc-6246, 1:500, respectively, Santa Cruz), pH2Ax, pFAK, FAK (05-636, 1:500; 05-1140, 1:500; 05-537, 1:1000 respectively, Millipore), Palladin (NBP1-25959, 1:1000, Novus Biologicals), SMAD3, SMAD2 and phosphoSMAD2/3 (9523, 3122, 3108 respectively; all 1:500, Cell Signalling), Collagen1 (ab138492 1:5000. Abcam).

TGF-β1 bioassay

TGF-β1 activation assays were performed co-culturing HFFF2 fibroblasts with MLEC cells stably expressing a TGF-β1-responsive luciferase reporter construct for 24 hours. Cells were lysed in reporter lysis buffer (Promega) and luciferase substrate was added to the supernatant. Luminescence was measured using a VarioSkan Flash plate reader (Perkin Elmer) and normalized with HFFF2s protein concentration [8].

Real-time PCR

RNA was extracted using the RNeasy mini-kit (Qiagen) and RNA samples were then reverse transcribed using the high capacity cDNA reverse transcription kit (Applied Biosytems) following the manufacturer's instructions. Real time PCR was performed using a POWER SYBR green mastermix (Applied Biosystems) plus 10ng of cDNA. The RT-PCR reaction was carried out with a 95°C hold step for 10 minutes to activate the polymerase, followed by 40 cycles of 95-60°C for 15 seconds and 5 minutes respectively. The mRNA expression levels were analyzed using the ΔΔCt relative quantitation method. Primers were designed using the http://www.ncbi.nlm.nih.gov/tools/primer-blast/. To avoid genomic DNA contamination at least one of the forward or the reverse primer annealed with an exon-exon junction [DNAse was also performed during the RNA extraction (Qiagen)]. Primers were tested for their efficiency using standard quantitation analysis accordingly to Applied Biosystems instructions. Primers sequences are in Supplementary Table S7.

Collagen gel contraction assays

$5x10^5$ fibroblasts were added to 1ml of ice-cold 3mg/ml type 1 collagen (Millipore) gels containing 10% FBS DMEM. Gels were incubated at 37C for 1-hour to polymerize. After adding 1ml of 10% FBS DMEM, gels were detached using a spatula and photographed after 24-hours incubation. Gel area was measured using Image J software.

Fibroblast Derived Matrices (FDMs)

30,000 HFFF2s were seeded to chamber slides or Transwell culture inserts coated with 0.2% gelatin to achieve a confluent monolayer. These cells were then cultured for 7-days in DMEM supplemented with 292μg/ml L-Glutamine, 10% FCS and 50μg/ml ascorbic acid, culture medium was replaced every 48-hours. Matrices were then visualized by immunofluorescence staining for Fibronectin or Collagen V, as described above.

For Tanswell invasion assay on FDMs the matrices were decellularized by incubation in PBS plus EDTA (50mM) and TritonX-100 (0.5% *v/v*) for 30 mins at 37°C followed by 1 hour incubation with DNAse (20U/ml) at room. Removal of cells using this technique was confirmed by staining for DAPI and phalloidin (data not shown). Decellularized FDMs were stored in PBS plus Penicillin/streptomycin (1% *v/v*) at 4ºC and washed with DMEM plus 10% FCS prior to use in Transwell invasion assays [64, 46]. For Collagen V staining, FDMs were decellularized by incubating with 0.25M NH4OH in 50mM Tris at 37ºC for 30mins, washed with PBS and then fixed with ice cold methanol at -20ºC for 30 mins before proceeding with the collagen V staining.

RNA-sequencing analysis

HFFF2 were treated 7-days prior to RNA extraction, RT-PCR and RNA-sequencing analysis. RNA-sequencing libraries were prepared for each RNA sample, sequenced using an Illumina HiSeq 2500 platform. The protocol employed yielded 35-bp long reads at an average sequencing depth of 42.2 million raw sequencing reads per sample (range 37.4-48.5 million). Of these reads, an average of 87.9% aligned to the hg19 reference genome file (range 85.6%-89.7%). The mappings were then converted to gene specific read count values using the script HTSeq-count [65, 66], yielding read count values for a total of 23,368 annotated genes. Differential expression analysis was performed in the Bioconductor Package EdgeR importing raw counts obtained by HTSeq [65, 66]. From these we excluded all genes that had less than 3 read counts, giving a total of 13,094 genes. The trimmed mean of M-values (TMM) was implemented to adjust for RNA composition, followed by estimation of the biological coefficient of variation (BCV). After negative binomial model were fitted and dispersion estimates obtained, differential expression analysis was performed using generalized linear model (GLM) likelihood ratio test.

Second Harmonic Generation Microscopy

Collagen fiber elongation was measured as follows, 512x512 pixel images (457μm^2 area, 20X Magnification) of stromal regions within Tissue Microarray cores (3-6 per patient) or xenograft tumors (3 ROIs per tumor) were imaged. CT-FIRE segmentation was then used to measure individual fiber lengths. As described previously [14], the presence of elongated collagen fibers within the matrix manifested in increased variance of fiber lengths due to preponderance for relatively small fibers, therefore the standard deviation was used as a measure of this change in collagen morphology and the mean value for each patient or xenograft was calculated.

Prognostic correlation testing

For categorical prognostic correlation testing the maximal Youden's index (Sensitivity + Specificity -1) for classifying patients with short survival rates (cancer specific survival < 3 years) was used as a cut-off value, as described previously [14].

TCGA

The retrospective collection cohort from the HNSCC TCGA was used to determine correlations between gene expression and patient survival. First patients were classed as positive for the presence of a myofibroblastic stroma, defined by higher than average expression of genes associated with myofibroblasts (ACTA2, CDH2, PDGFRA, PDGFRB, VIM, S100A4). Within this group of patients the correlation of individual genes or gene signatures (mean value for the expression of each gene within the signature) with survival was then tested using Kaplan-Meier survival analysis and Log-Rank (Mantel-Cox) tests. The primary endpoint was death with evidence of tumor as disease specific survival was not available for this database, deaths without evidence of tumor were censored at the time of death.

Supplementary Table S1. Immunohistochemistry analysis of myofibroblast and senescence marker co-expression in the tumor stroma.

Cancer Type	Cases	SMA High/Mod Stroma (Co-expression of p21/p53)	SMA Low Stroma (Co-expression of p21/p53)
**EAC**	21	16 (2)	5 (0)

Supplementary Table S2. GO analysis of TGF-β1 or IR DEGs using GSEA Molecular Signature Database (MSigDB) (20 most significantly up/down-regulated GO terms).

**TGF-β1 Up-regulated**				
**GO TERM**	**SIZE**	**NES**	**NOM p**	**FDR q**
PROTEINACEOUS EXTRACELLULAR MATRIX	72	2.001	<0.001	0.067
EXTRACELLULAR MATRIX	73	1.998	<0.001	0.035
SKELETAL DEVELOPMENT	71	1.902	<0.001	0.094
INTERPHASE OF MITOTIC CELL CYCLE	57	1.846	<0.001	0.140
DNA REPLICATION	95	1.817	<0.001	0.159
MUSCLE DEVELOPMENT	73	1.780	0.002	0.203
EXTRACELLULAR MATRIX STRUCTURAL CONSTITUENT	20	1.767	0.002	0.201
BRAIN DEVELOPMENT	32	1.763	0.000	0.182
SKELETAL MUSCLE DEVELOPMENT	22	1.763	0.011	0.163
COLLAGEN	20	1.744	0.014	0.181
ACTIN FILAMENT ORGANIZATION	17	1.702	0.017	0.249
MYOBLAST DIFFERENTIATION	15	1.693	0.009	0.246
REGULATION OF GROWTH	47	1.692	0.004	0.229
ORGAN DEVELOPMENT	352	1.677	0.000	0.245
POTASSIUM CHANNEL ACTIVITY	16	1.672	0.020	0.238
STRIATED MUSCLE DEVELOPMENT	31	1.663	0.015	0.243
DNA METABOLIC PROCESS	233	1.659	0.000	0.238
PROTEIN COMPLEX BINDING	37	1.655	0.007	0.234

DNA REPAIR	116	1.614	0.002	0.312
REGULATION OF CELL GROWTH	40	1.602	0.013	0.329
**TGF-β1 Down-Regulated**				
**GO TERM**	**SIZE**	**NES**	**NOM p**	**FDR q**
REGULATION OF BODY FLUID LEVELS	26	-2.210	<0.001	0.006
HEMOSTASIS	23	-2.174	<0.001	0.005
WOUND HEALING	25	-2.157	<0.001	0.004
STEROID METABOLIC PROCESS	39	-2.154	<0.001	0.003
BLOOD COAGULATION	19	-2.083	<0.001	0.005
LOCOMOTORY BEHAVIOR	45	-2.051	<0.001	0.008
COAGULATION	19	-2.050	<0.001	0.007
CHEMOKINE RECEPTOR BINDING	21	-2.039	<0.001	0.007
HEMATOPOIETIN INTERFERON CLASSD200 DOMAIN CYTOKINE RECEPTOR ACTIVITY	17	-2.020	<0.001	0.008
CHEMOKINE ACTIVITY	21	-2.013	0.005	0.008
VESICULAR FRACTION	29	-1.983	<0.001	0.012
CELL CELL SIGNALLING	188	-1.970	<0.001	0.013
MICROSOME	27	-1.958	0.002	0.014
EXTRACELLULAR SPACE	107	-1.946	<0.001	0.015
G PROTEIN COUPLED RECEPTOR BINDING	24	-1.920	0.002	0.018
BEHAVIOR	61	-1.917	0.002	0.018
LIPID RAFT	19	-1.899	<0.001	0.022
HYDROLASE ACTIVITY HYDROLYZING O GLYCOSYL COMPOUNDS	24	-1.889	<0.001	0.023
CYTOKINE BINDING	28	-1.879	<0.001	0.024
CYTOKINE ACTIVITY	54	-1.841	<0.001	0.035

IR Up-regulated				
**GO TERM**	**SIZE**	**NES**	**NOM p**	**FDR q**
CONTRACTILE FIBER PART	15	2.149	<0.001	0.013
CONTRACTILE FIBER	16	2.067	<0.001	0.021
REGULATION OF PROTEIN AMINO ACID PHOSPHORYLATION	18	1.893	<0.001	0.117
EXTRINSIC TO MEMBRANE	15	1.870	<0.001	0.111
STRUCTURAL CONSTITUENT OF MUSCLE	18	1.821	0.013	0.161
ANION TRANSMEMBRANE TRANSPORTER ACTIVITY	21	1.816	0.005	0.142
BASOLATERAL PLASMA MEMBRANE	27	1.811	0.003	0.126
POSITIVE REGULATION OF PHOSPHATE METABOLIC PROCESS	20	1.745	0.008	0.214
PHOSPHOLIPID BIOSYNTHETIC PROCESS	37	1.743	0.003	0.191
PHOSPHOINOSITIDE BIOSYNTHETIC PROCESS	24	1.737	0.003	0.182
POSITIVE REGULATION OF PHOSPHORYLATION	18	1.725	0.011	0.183
STEROID METABOLIC PROCESS	39	1.724	0.009	0.169
MITOCHONDRIAL RESPIRATORY CHAIN	23	1.711	0.008	0.174
MITOCHONDRIAL MEMBRANE PART	50	1.709	0.003	0.164
PHOSPHOLIPID METABOLIC PROCESS	60	1.700	<0.001	0.165
INTRINSIC TO ENDOPLASMIC RETICULUM MEMBRANE	20	1.683	0.006	0.181
LIPOPROTEIN BIOSYNTHETIC PROCESS	23	1.674	0.012	0.185
INTEGRAL TO ENDOPLASMIC RETICULUM MEMBRANE	20	1.671	0.009	0.178
PROTEIN AMINO ACID LIPIDATION	23	1.664	0.019	0.181
GLYCEROPHOSPHOLIPID BIOSYNTHETIC PROCESS	28	1.636	0.012	0.214

IR Down-Regulated				
**GO TERM**	**SIZE**	**NES**	**NOM p**	**FDR q**
M PHASE OF MITOTIC CELL CYCLE	79	-2.482	<0.001	<0.001
MITOSIS	76	-2.458	<0.001	<0.001
MITOTIC CELL CYCLE	142	-2.361	<0.001	<0.001
M PHASE	100	-2.346	<0.001	<0.001
CELL CYCLE PROCESS	174	-2.300	<0.001	<0.001
CHROMOSOMAL PART	88	-2.293	<0.001	<0.001
CELL CYCLE PHASE	151	-2.242	<0.001	<0.001
CHROMOSOME	114	-2.240	<0.001	<0.001
SPINDLE	38	-2.170	<0.001	<0.001
REGULATION OF MITOSIS	37	-2.138	<0.001	<0.001
CELL CYCLE GO 0007049	286	-2.117	<0.001	<0.001
CHROMOSOMEPERICENTRIC REGION	31	-2.092	<0.001	<0.001
CENTROSOME	54	-2.064	<0.001	<0.001
CHROMOSOME SEGREGATION	31	-2.058	<0.001	<0.001
MITOTIC SISTER CHROMATID SEGREGATION	15	-2.028	<0.001	<0.001
CHROMOSOME ORGANIZATION AND BIOGENESIS	112	-2.023	<0.001	0.001
MICROTUBULE CYTOSKELETON	138	-2.015	<0.001	0.001
ESTABLISHMENT OF ORGANELLE LOCALIZATION	15	-2.004	<0.001	0.001
MICROTUBULE ORGANIZING CENTER	62	-2.003	<0.001	0.001
SISTER CHROMATID SEGREGATION	16	-2.002	<0.001	0.001

Supplementary Table S3. Analysis of expression changes in collagen super-family genes in TGF-β1 and IR treated fibroblasts from RNA-sequencing data.

Fibrillar Collagens				
**Collagen Type**	**TGF-β1**		**IR**	
	Log2 FC	adj p	Log2 FC	adj p
COL1A1	1.421861	2.24E-18	-0.453	0.009147
COL1A2	0.472091	0.000124	-0.178	0.179794
COL2A1			-0.951	9.87E-14
COL3A1	0.355003	0.00163	-0.348	3.13E-05
COL5A1	1.07646	6.68E-29	-0.190	0.036575
COL5A2	0.381071	0.000467	-1.818	1.33E-21
COL5A3	0.814222	6.21E-08	-0.355	0.007368
COL6A1	0.208728	0.471917	-0.445	0.045791
COL6A2	0.328719	0.415088	-0.947	7.39E-37
COL6A3	0.687195	3.75E-12	-0.328	0.979024
COL24A1	0.880823	2.43E-06	-0.086	1
COL27A1	0.588034	0.000227	-1.249	0.178365
**Non-Fibrillar Collagens**				
**Collagen Type**	**TGF-β1**		**IR**	
	Log2 FC	adj p	Log2 FC	adj p
COL4A1	1.465475	6.05E-53	-0.434	0.002008
COL4A2	1.309765	1.01E-19	-0.337	0.940539
COL4A3	-0.5921	0.779248	0.245	0.031401
COL4A4	0.912166	0.023803	-0.274	0.011552
COL4A5	-0.55361	1.92E-06	-1.282	9.96E-13

COL4A6	-2.35057	8.24E-26	-0.557	1.89E-10
COL7A1	0.945709	7.66E-05	0.742	1.19E-20
COL8A1	0.084103	0.686296	-0.074	0.776211
COL8A2	1.481366	1.10E-36	-0.989	0.061695
COL9A2	0.317961	0.614686	-1.405	0.129875
COL10A1	1.286729	4.39E-06	0.169	0.872063
COL11A1	2.113575	2.64E-45	1.282	1.01E-31
COL11A2	0.774193	0.027028	0.294	0.666247
COL12A1	0.214727	0.118621	-0.072	0.815616
COL13A1	-0.19034	0.305731	0.086	0.790883
COL14A1	-1.17541	8.69E-31	-1.745	1.85E-89
COL15A1	0.54311	0.012655	-2.145	1.36E-14
COL16A1	0.933687	3.39E-10	-0.251	0.103717
COL17A1	0.347334	0.877019	-1.273	0.378297
COL18A1	-0.28334	0.434086	-0.695	1.74E-05
COL19A1	-0.14401	1	0.983	0.597306
COL21A1	-0.81087	4.22E-08	-2.543	5.05E-53
COL25A1	0.779882	0.222591	-0.110	0.710796
COL28A1	-0.35078	0.7906		

Significant up-regulations (adjusted P value <0.001) are highlighted in red and significant down-regulations are highlighted in blue.

Supplementary Table S4. KME values for the collagen genes in the HNSCC WGCNA.

Id	SE.ECM	SE.TGFB	SE.IR	SE.Verrecchia_TGFB	SE.Fridman_senes
COL17A1	0.09	0.22	0.65	0.49	0.68
COL4A6	0.11	0.20	0.59	0.46	0.58
COL13A1	0.41	0.49	0.52	0.68	0.64
COL12A1	0.71	0.77	0.49	0.82	0.63
COL16A1	0.45	0.56	0.48	0.69	0.61
COL5A3	0.73	0.78	0.47	0.82	0.62
COL5A1	0.83	0.89	0.41	0.88	0.60
COL5A2	0.88	0.91	0.35	0.86	0.53
COL4A1	0.69	0.72	0.33	0.77	0.49
COL4A2	0.64	0.68	0.31	0.76	0.49
COL6A1	0.89	0.89	0.23	0.80	0.44
COL1A1	0.92	0.92	0.23	0.80	0.42
COL6A2	0.89	0.89	0.21	0.79	0.42
COL1A2	0.94	0.92	0.20	0.78	0.39
COL18A1	0.70	0.71	0.18	0.67	0.35
COL3A1	0.94	0.92	0.17	0.76	0.36
COL6A3	0.94	0.91	0.14	0.76	0.34
COL24A1	0.84	0.82	0.11	0.65	0.26
COL11A1	0.78	0.75	0.06	0.60	0.22
COL8A1	0.87	0.82	-0.02	0.65	0.17
COL8A2	0.62	0.61	-0.03	0.44	0.13
COL15A1	0.70	0.66	-0.04	0.53	0.13
COL10A1	0.81	0.77	-0.06	0.56	0.12
COL14A1	0.67	0.62	-0.08	0.41	0.04

Supplementary Table S5. Summary of the number of SMA positive HNSCC and EAC tumors with short or elongated collagen fibers.

Collagen Fiber Length	HNSCC			EAC		
	N	Events	Censored	N	Events	Censored
Low	30	5	83.3%	53	20	62.3%
High	83	34	59.0%	68	41	39.7%
Total	113	39	65.5%	121	61	49.6%

Supplementary Table S6. Table of clinico-pathological features.

		HNSCC	EAC
**Age**	Median (min, max)	61 (28, 90)	70 (41, 89)
**Sex**	Male	80	103
	Female	33	18
**T Stage**	T0		7
	T1	25	24
	T2	46	32
	T3	12	55
	T4	29	3
**N Stage**	N0	27	61
	N1	12	29
	N2	67	17
	N3	6	14
**M Stage**	M0	112	116
	M1	1	5
**Grade**	Well Differentiated	1	11
	Mod. Differentiated	42	34
	Poorly Differentiated	70	75
	Undifferentiated		1

Supplementary Table S7. Real Time PCR primers sequence.

Gene	Forward	Reverse
COL1A1	ACGAAGACATCCCACCAATCACCT	AGATCACGTCATCGCACAACACCT
COL3A1	AATCAGGTAGACCCGGACGA	TTCGTCCATCGAAGCCTCTG
CTGF	CCCTCGCGGCTTACCGACTG	GGCGCTCCACTCTGTGGTCT
MMP2	GAACTTCCGTCTGTCCCAGG	GTCAGGAGAGGCCCCATAGA
CDKN1A	CTGGAGACTCTCAGGGTCGAA	CCAGGACTGCAGGCTTCCT
FN1	TGTGGTTGCCTTGCACGA	GCTTGTGGGTGTGACCTGAGT
GAPDH	AGCAATGCCTCCTGCACCACCAAC	CCGGAGGGGCCATCCACAGTCT
ACTA2	GACAATGGCTCTGGGCTCTGTAA	ATGCCATGTTCTATCGGGTACTT
COL1A2	GGCCCTCAAGGTTTCCAAGG	CACCCTGTGGTCCAACAACTC
SERPINE1/PAI1	CACAACCCCACAGGAACAGT	ATGAAGGCGTCTTTCCCCAG

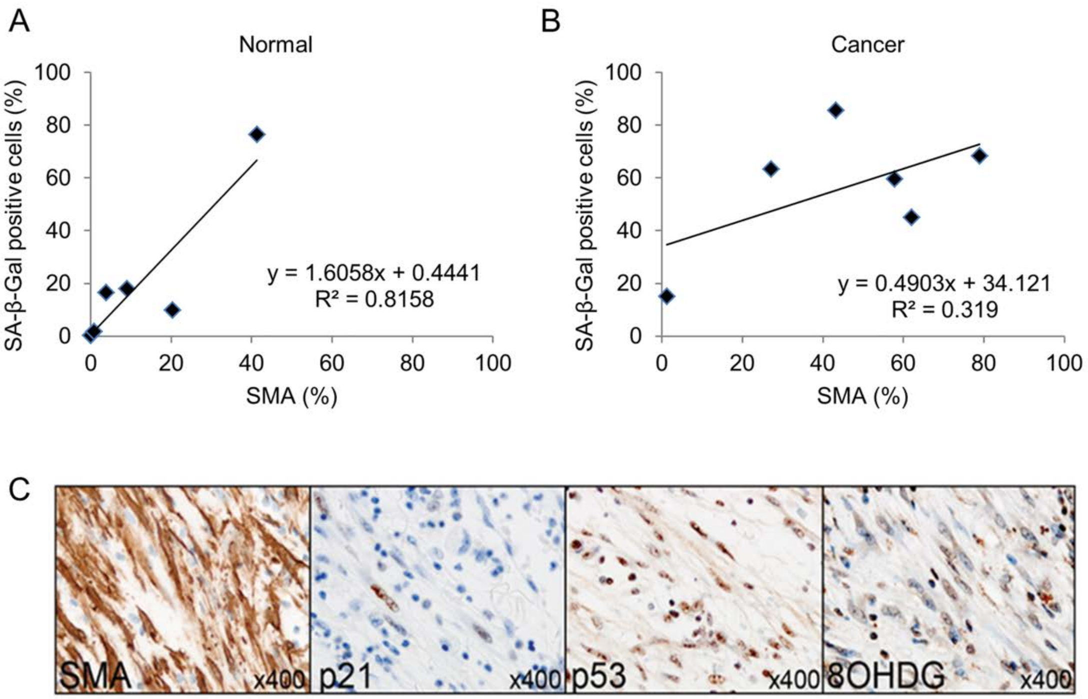

Supplementary Figure S1. Senescent CAF isolated ex vivo and analyzed in vivo are mostly SMA-positive. (**A**-**B**) Graphs showing the linear regression analysis of the percentage of cells positive for senescence-associated (SA)-β-Galactosidase or SMA stress fiber positivity in normal oral fibroblasts (POF) (**A**) and cancer-associated oral fibroblasts (CAF) (**B**) of Fig. 1A. (**C**) Representative images of immunohistochemical staining for SMA and markers of senescence (p53, p21 and 8-OHDG) on sequential tissue sections of esophageal adenocarcinomas (EAC).

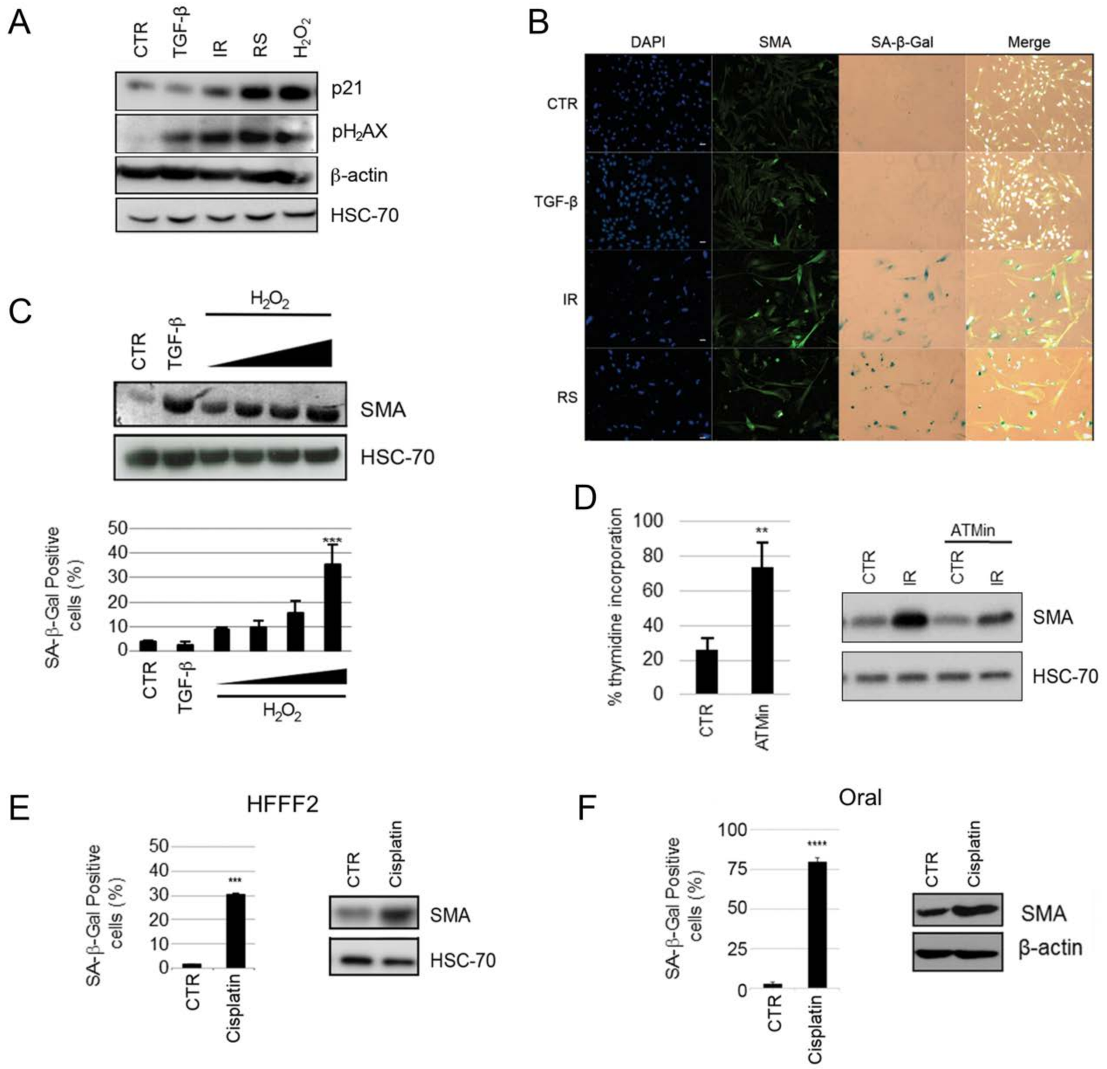

Supplementary Figure S2. Induction of fibroblast senescence generates a myofibroblastic phenotype. HFFF2s were treated as described in Fig. 2 (**A-E**). Senescence was confirmed by Western blot for expression of p21 and pH2Ax proteins (HSC-70 as loading control; (**A, B**) SMA immunofluorescence (green) and SA-β-galactosidase staining (blue) showing co-localization in HFFF2 fibroblasts induced to senesce as indicated (Scale Bar represents 100µm); (**C**) Dose-dependent effect of H_2O_2 treatment (single treatment with 0.1, 0.2, 0.5 and 1mM) on expression of SMA (HSC-70 as loading control; top panel) and SA-β-Galactosidase quantification (bottom panel); (**D**) Inhibition of irradiation driven senescence and myofibroblast differentiation in HFFF2 treated with 20µM ATM inhibitor (ATMin); 16 hours thymidine incorporation 5 days after 10Gy γ-irradiation expressed in percentage compared to the untreated cells (left panel) and western blot for SMA (HSC-70 as loading control; right panel); (**E-F**) Cisplatin induction of senescence (SA-β-Galactosidase assay; left panels) and SMA expression (western blot; right panels) in HFFF2 (**E**) and normal primary oral fibroblasts (**F**). Data are presented as mean ± SEM and statistics are shown for T-test compared to controls (*p<0.05, **p<0.01, ***p<0.001, ****p<0.0001).

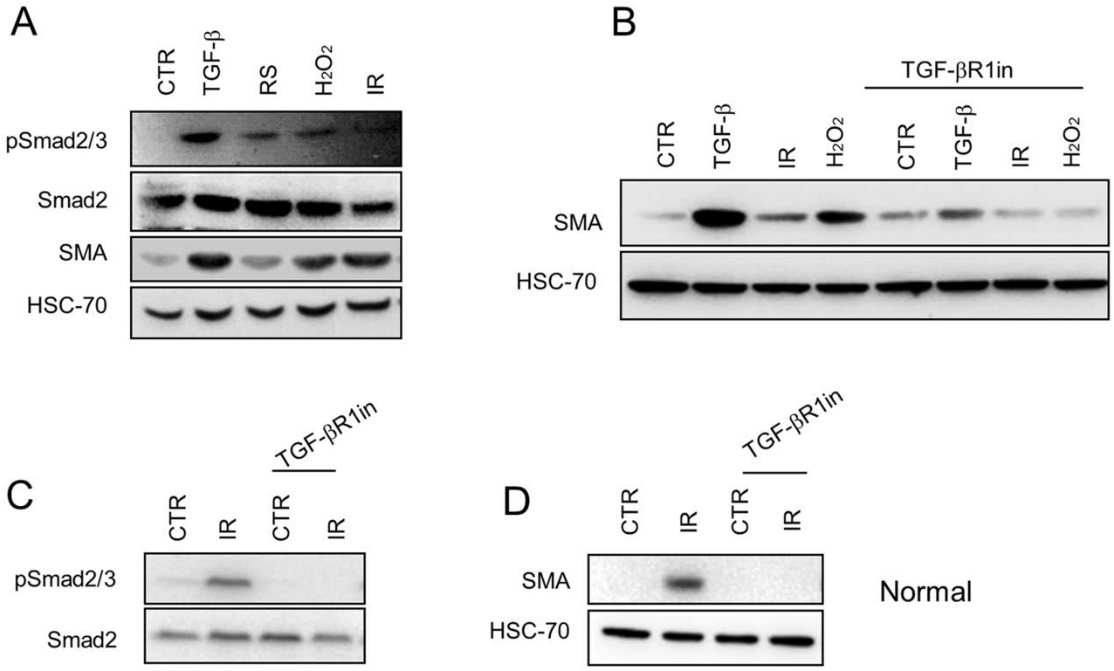

Supplementary Figure S3. Smad signaling mediates senescence induction of a myofibroblastic phenotype. (**A**) Western blot showing phospho(p)-Smad2/3 expression in HFFF2 fibroblasts treated as described in the figure (Smad2 and HSC-70 as loading controls); (**B**) Western blot for SMA of HFFF2 fibroblasts treated with TGF-β1, IR or H_2O_2 in presence of TGF-βR1 inhibitor (HSC-70 as loading control; (**C**-**D**) HFFF2 cells were pre-treated with TGF-βR1 inhibitor and irradiated: (**C**) Western blot for pSmad2/3 two hours after irradiation (Smad2 as loading controls); (**D**) Western blot for SMA four days after irradiation (HSC-70 as loading control).

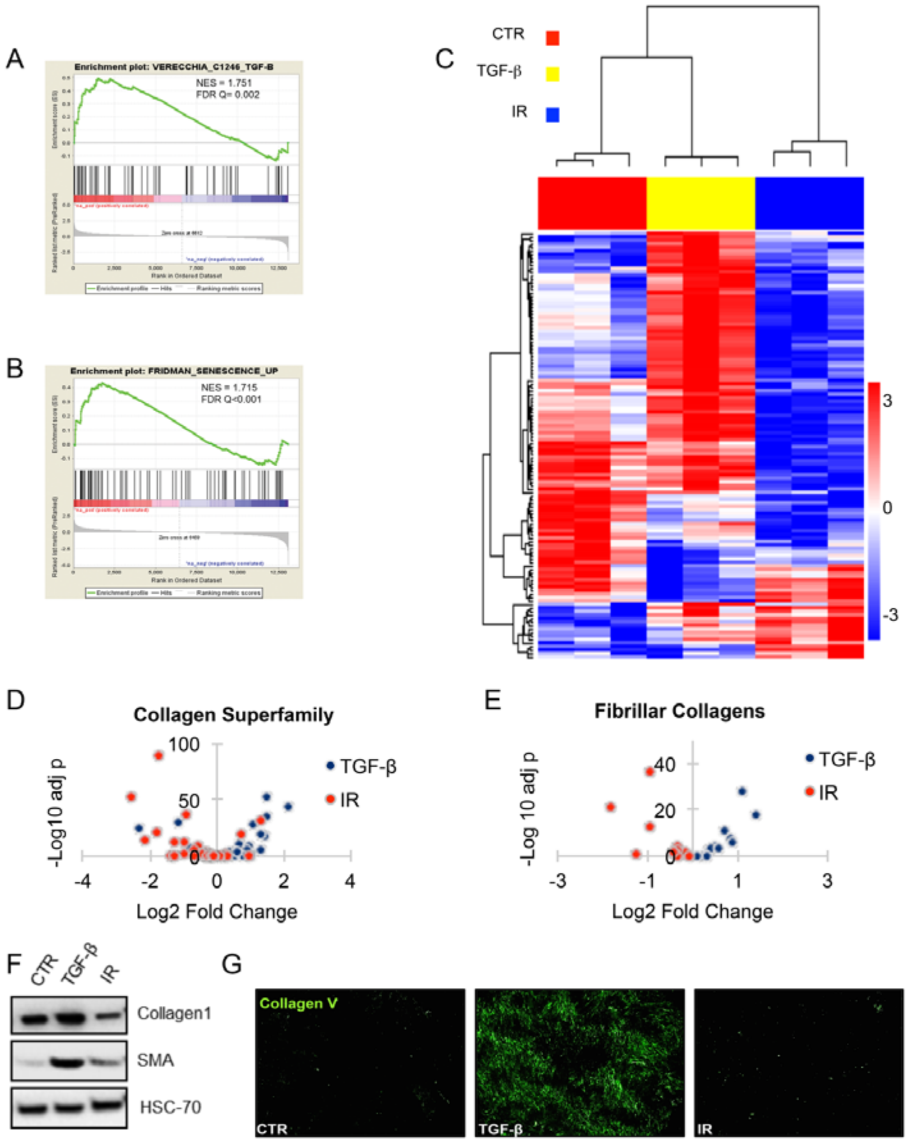

Supplementary Figure S4. Senescent fibroblasts and myofibroblasts have divergent gene expression. RNA-sequencing analysis of HFFF2 cells treated with IR or TGF-β1 and grown for 7 days (**A-D**). (**A-B**) Gene set enrichment analysis (GSEA) of a signature associated with TGF-β1 treatment (**A**) and senescence (**B**) compared with either TGF-β1 or IR DEGs respectively. (**C**) Unsupervised hierarchical clustering of DEGs from RNA-sequencing analysis of HFFF2s within the core matrisome dataset showing predominant up-regulation of ECM genes in TGF-β1 treated fibroblasts compared to down-regulation in IR treated fibroblasts. Expression levels were subjected to Z score scaling within each sample for visualization purposes. Distances were calculated using a Euclidean distance measure. (**D-E**) Volcano plots showing the fold change and significance (FDR adj p-value) of genes belonging to the collagen superfamily (**D**), and fibrillar collagens (**E**) present in the TGF-β1 and IR treated HFFF2s of the RNA-sequencing experiment. (**F**) Western blot showing inhibition of collagen 1 expression in IR-treated fibroblasts compared with TGF-β1-treated cells (HSC-70 as loading control). Western confirms upregulation of SMA. Representative images of immunofluorescence of HFFF2 decellularized FDM showing weak expression of Collagen V (green) in irradiated fibroblasts compared with TGF-β1-treated cells.

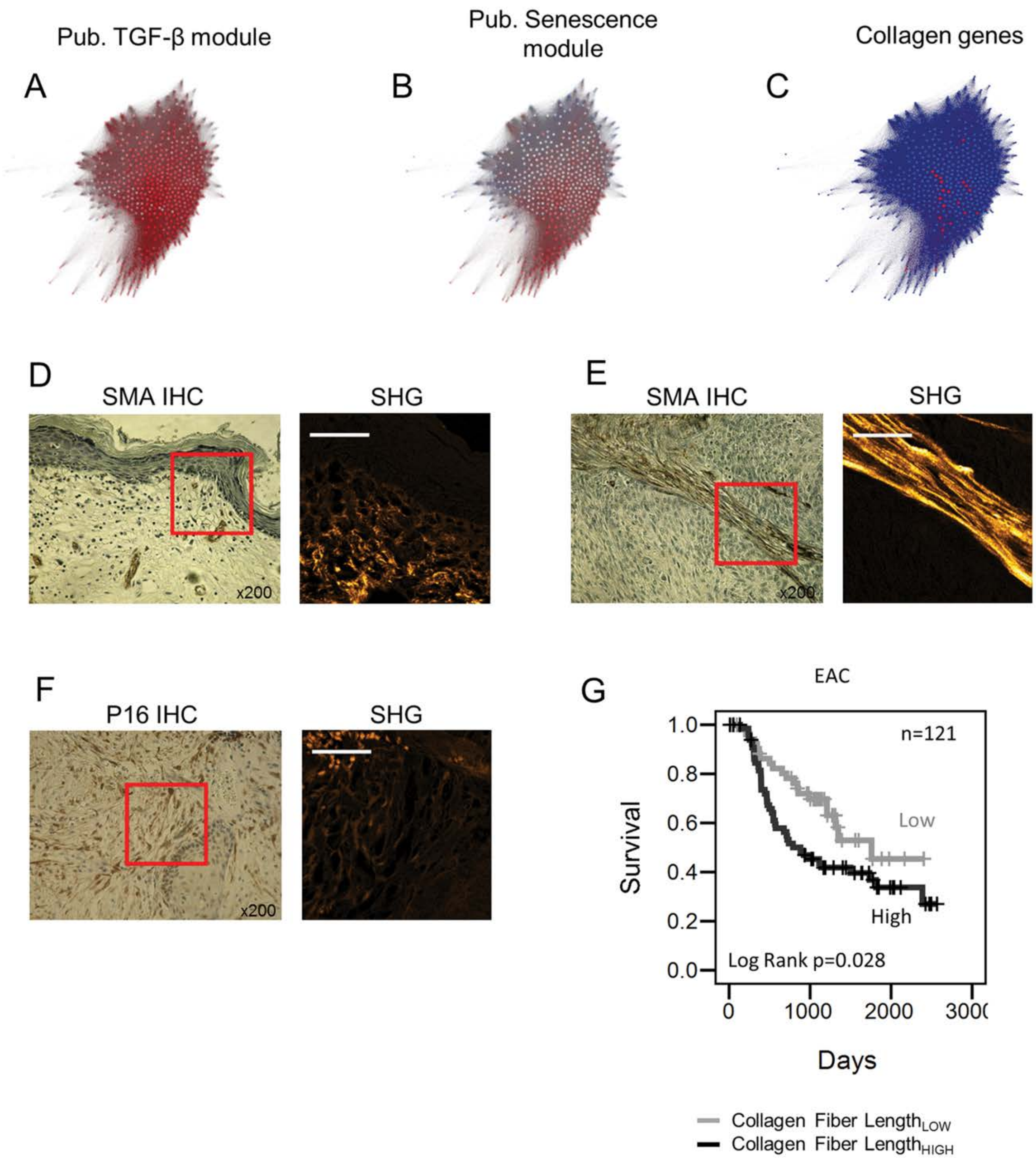

Supplementary Figure S5. Collagen fiber deposition impacts tumor progression. (**A-C**) Network graphs of the ECM module where nodes are color coded according to the correlation with a publicly available TGF-β (**A**) or senescence geneset (**B**); red and blue colors show positive and negative correlation, respectively; (**C**) Network graphs of the ECM module where the red nodes indicate the collagen genes. (**D-F**) Representative images of SMA immunohistochemistry staining (left) or Second Harmonic Generation imaging (right) from the same tissue sections. (**D**) Normal oral squamous epithelium. (**E**) SMA-positive/p16-negative HNSCC. (**F**) Representative image of p16 immunohistochemistry staining (left) or Second Harmonic Generation imaging (right) from the same tissue sections of an SMA-positive/p16-positive HNSCC case (Scale Bars indicate 100µm). (**G**) Kaplan-Meier curves showing disease specific survival (DSS) rates in EAC patients with moderate or high stromal SMA expression (measured by immunohistochemistry), stratified by collagen fiber elongation measured by Second Harmonic Generation imaging.
